# Treatment of rice straw hemicellulosic hydrolysates with advanced oxidative processes: a new and promising detoxification method to improve the bioconversion process

**DOI:** 10.1186/1754-6834-6-23

**Published:** 2013-02-15

**Authors:** João Paulo Alves Silva, Livia Melo Carneiro, Inês Conceição Roberto

**Affiliations:** 1 Deparatmento de Biotecnologia, Escola de Engenharia de Lorena, Universidade de São Paulo, Estrada Municipal do Campinho s/n, Cep, 12602-810, Lorena, SP, Brazil

**Keywords:** Ethanol, *Pichia stipitis*, Rice straw hemicellulosic hydrolysate, Advanced oxidative processes, Detoxification

## Abstract

**Background:**

The use of lignocellulosic constituents in biotechnological processes requires a selective separation of the main fractions (cellulose, hemicellulose and lignin). During diluted acid hydrolysis for hemicellulose extraction, several toxic compounds are formed by the degradation of sugars and lignin, which have ability to inhibit microbial metabolism. Thus, the use of a detoxification step represents an important aspect to be considered for the improvement of fermentation processes from hydrolysates. In this paper, we evaluated the application of Advanced Oxidative Processes (AOPs) for the detoxification of rice straw hemicellulosic hydrolysate with the goal of improving ethanol bioproduction by *Pichia stipitis* yeast. Aiming to reduce the toxicity of the hemicellulosic hydrolysate, different treatment conditions were analyzed. The treatments were carried out according to a Taguchi L_16_ orthogonal array to evaluate the influence of Fe^+2^, H_2_O_2_, UV, O_3_ and pH on the concentration of aromatic compounds and the fermentative process.

**Results:**

The results showed that the AOPs were able to remove aromatic compounds (furan and phenolic compounds derived from lignin) without affecting the sugar concentration in the hydrolysate. Ozonation in alkaline medium (pH 8) in the presence of H_2_O_2_ (treatment A3) or UV radiation (treatment A5) were the most effective for hydrolysate detoxification and had a positive effect on increasing the yeast fermentability of rice straw hemicellulose hydrolysate. Under these conditions, the higher removal of total phenols (above 40%), low molecular weight phenolic compounds (above 95%) and furans (above 52%) were observed. In addition, the ethanol volumetric productivity by *P. stipitis* was increased in approximately twice in relation the untreated hydrolysate.

**Conclusion:**

These results demonstrate that AOPs are a promising methods to reduce toxicity and improve the fermentability of lignocellulosic hydrolysates.

## Background

Lignocellulosic materials represent one of the most promising sources of renewable raw material for various biotechnological processes due to their low economic value and high availability. Composed of polysaccharides (cellulose and hemicellulose) and lignin**,** these materials are rigid and fibrous in structure [1-4]. A selective separation of the main fractions are required to use these materials in biotechnological processes, which can be performed using different physical, chemical and biochemical pretreatment methods and various combinations thereof [5]. During acid hydrolysis, several toxic compounds are formed by the degradation of sugars and lignin. These compounds are mainly responsible for the toxicity of hemicellulosic hydrolysates due to their ability to inhibit microbial metabolism. Thus, the use of a detoxification step represents an important aspect to be considered for the improvement of fermentation processes from hydrolysates [6].

Several methods have been proposed to reduce the concentration of toxic compounds to levels that would not inhibit the fermentation process [7-9]. These methods can be divided into the following three main groups: biological, physical and chemical. Biological treatments involve the use of microorganisms or enzymes that act on the toxic compounds present in the hydrolysate by changing their chemical structures [8,10]. The physical methods promote the removal of toxic compounds from the medium without changing their chemical structures. These methods are based on adsorption processes and include the use of activated charcoal [11], ion exchange resins [12], diatomaceous earth [13], or phase transfer processes such as solvent extraction [14] and evaporation [9]. Currently, the main chemical detoxification methods employed in hydrolysates treatment are based on the addition of reductive substances and pH modification. Reductive substances may lead to chemical modifications of the toxic inhibitor compounds, thereby changing the degree of toxicity [15]. The processes based on pH modification lead to a reduction in hydrolysate toxicity by two different mechanisms. The first mechanism is due to the pH-dependent precipitation of toxic compounds, and the second method is attributed to the decomposition of certain compound inhibitors due to their chemical instability at certain pH values [16,17]. Many of the detoxification methods based on physical, chemical and biological processes can result in a considerable reduction to the sugar concentration; however these sugar´s losses are undesirable for fermentation purposes [18-21].

A new, promising and little studied method for the detoxification of lignocellulosic hydrolysates is the use of advanced oxidative processes (AOPs). AOPs have been studied for the remediation of lignin derivatives from pulp and paper industry wastewater [22]. Such processes have the ability to degrade toxic and recalcitrant compounds, thereby reducing the toxicity of effluents and enhancing their susceptibility to biological agents. Therefore, it is possible to apply AOPs as a method of reducing the toxicity of lignocellulosic hydrolysates. Advanced oxidative processes (AOPs) can be defined as those methods where hydroxyl radicals (HO•) are produced in sufficient quantities to act as the main oxidizing agent [23]. The hydroxyl radical is a powerful oxidizing agent that is able to mineralize biorecalcitrant organic compounds or convert them into biodegradable compounds [24]. Due to its high reactivity, the hydroxyl radical must be generated *in situ*, which may be accomplished with a number of different processes [25]. Hydroxyl radicals can be generated as a result of a combination of strong oxidizing agents, such as hydrogen peroxide and ozone. Ultraviolet (UV) or visible radiation and catalysts such as metal ions and semiconductors can also be used to create hydroxyl radicals [26,27]. The reactions between hydroxyl radicals and organic molecules present in the medium can be classified into the following three classes according to their reaction mechanisms: hydrogen atom extraction, electrophilic addition and electron transfer [28].

The prevalence of a particular type of reaction in organic compound oxidation depends on multiple factors, including the compound concentration and the chemical structure [26]. Generally, electrophilic addition reactions tend to occur more quickly than the subtraction of hydrogen atoms or electron transfer in aromatic compounds and unsaturated hydrocarbons. The electrophilic nature of the hydroxyl radical can lead to a preferential attack of compounds with aromatic rings, which have a high electron density [29,30].

The effluents from industrial cellulose pulps are composed mainly of phenolic and aromatic compounds resulting from the degradation of lignin and carbohydrates. Many authors have reported a reduction in toxicity and an increase in the biodegradability of different toxic pollutants through treatment by AOPs [31-33]. Although AOPs have been widely studied to reduce the toxicity of various types of industrial effluents, the use these methods to detoxify lignocellulosic hydrolysates is a field still unexplored; there being a great shortage of work in this area.

In this context, the present study aimed to evaluate AOPs as a method to detoxify rice straw hemicellulosic hydrolysates to improve the ethanol production by *Pichia stipitis*.

## Results and discussion

### Characterization of rice straw hemicellulosic hydrolysate

The Rice Straw Hemicellulosic Hydrolysate (RSHH) was obtained by dilute acid hydrolysis and concentrated by vacuum evaporation. The composition of the hydrolysate is shown in Table 1. As shown, the hydrolysate presented a total sugar concentration of approximately 160 g/L, with a ratio of 6:1:1 of xylose, glucose and arabinose. In addition to sugars (Table 1) compounds potentially harmful to the fermentation process, such as carboxylic acids, furans and phenolic compounds, were also identified. The RSHH contained approximately 2 g/L acetic acid, whose origin is attributed to the acetyl groups present in the hemicellulose polymers that are released during hydrolysis. The presence of 211 mg/L furfural and 116 mg/L hydroxymethylfurfural (HMF) in RSHH demonstrates the partial degradation of xylose and glucose, respectively.

**Table 1:** Composition of the rice straw hemicellulosic hydrolysate as its major constituents

**Components**	**Units**	**RSHH**
Total solids	(g/L)	305.0 ± 1.9
Ash	(g/L)	31.4 ± 0.6
Carbohydrates and aliphatic acids		
Xylose	(g/L)	115.9 ± 1.0
Glucose	(g/L)	20.8 ± 0.1
Arabinose	(g/L)	21.2 ± 0.2
Acetic acid	(g/L)	1.96 ± 0.01
Furans		
Furfural	(mg/L)	211 ± 4
Hydroxymethylfurfural	(mg/L)	116 ± 10
Phenolic compounds		
Total phenolic compounds	(g/L)	12.9 ± 0.8
Low molecular weight phenolic compounds		
Ferulic acid	(mg/L)	270 ± 8
*p-*coumaric acid	(mg/L)	106 ± 2
Syringic acid	(mg/L)	53 ± 3
Vanillic acid	(mg/L)	32 ± 4
Vanillin	(mg/L)	28 ± 6
Hydroxybenzoic acid	(mg/L)	14 ± 3
Minerals		
Sulfate	(mg/L)	9998 ± 1195
K	(mg/L)	5779 ± 233
Fe	(mg/L)	1765 ± 99
Mn	(mg/L)	1217 ± 25
Mg	(mg/L)	923 ± 40
Phosphate	(mg/L)	927 ± 99
Cr	(mg/L)	332 ± 50
Al	(mg/L)	266 ± 23
Na	(mg/L)	195 ± 36
Ca	(mg/L)	137 ± 58
Zn	(mg/L)	30 ± 4
Cu	(mg/L)	0.4 ± 0.2

Chemical compositions of rice straw hemicellulosic hydrolysate obtained after concentration process (RSHH).

The rice straw hemicellulosic hydrolysate had a high concentration of phenolic compounds (approximately 12.9 g/L) compared to acetic acid (approximately 2 g/L) and furan (327 mg/L). Only 4% of the total phenolic compounds present in RSHH were identified by HPLC (Table 1). In fact, the chromatographic technique only permits the identification of monomeric compounds. It is likely that 96% of the total phenol content can be attributed to polyphenolic structures. Among the compounds derived from the solubilized lignin in acidic medium, the hydrolysate contained vanillin (28 mg/L), *p-*coumaric acid (106 mg/L), vanilinic acid (32 mg/L), syringic acid (53 mg/L), ferulic acid (270 mg/L) and p-hydroxybenzoic acid (14 mg/L). These compounds have been suggested to be potential inhibitors to microbial metabolism [34].

### Detoxification treatment

Aiming to evaluate the potential application of AOPs in reducing the toxicity of rice straw hemicellulosic hydrolysate, assays were conducted to study the influence of Fe^+2^, H_2_O_2_, UV irradiation, O_3_ and pH. Each variable and combinations thereof was studied in terms of its ability to reduce the concentration of phenolic compounds (total and low molecular weight) and furans. The *Pichia stipitis* fermentability of the treated hydrolysates was also assessed for each condition.

### The effect of treatment on the sugar concentration

There was no significant change in the concentration of sugars for all of the conditions evaluated (data not shown). This is a positive result; methods of detoxification based on physical, chemical and biological treatment often results in a considerable reduction in the concentration of sugars [18,19,21]. For example, Carvalho *et al.*[35] evaluated the detoxification of sugarcane bagasse hemicellulosic hydrolysate by activated charcoal and a combined system of activated carbon and ion exchange resin. The authors observed a 19% and 40% reduction in the xylose concentrations with activated carbon and the combined system, respectively. Mussatto, Santos and Roberto [21] evaluated the detoxification process for a rice straw hemicellulosic hydrolysate using activated carbon adsorption and observed that, the xylose concentrations were reduced by 4% to 18%. In the present study, the low influence of the treatments by AOPs on the carbohydrates concentration may be related to the molecular structure of the sugars. The hydroxyl radical, the main oxidizing agent of AOPs, has a higher affinity for compounds with regions of high electron density, such as aromatic rings [29,30]. Such structures do not exist in sugar molecules.

### The effect of treatment on the concentration of phenolic and furan compounds

Table 2 shows the percent reduction in the concentration of total furans (furfural and HMF), low molecular weight phenolic compounds (LMWPC) and total phenolic compounds after hydrolysate treatment. Hydrolysates were treated according to the assay conditions specified by the experimental design of Taguchi.

**Table 2:** Experimental design and results obtained in the detoxification treatment and during the fermentation of hydrolysate treated

**Assays**	**Experimental conditions (Real values)**															**Reduction (%)**			**Q**_P _** (%)
	**A**	**B**	**AB**	**C**	**AC**	**BC**	**DE**	**D**	**AD**	**BD**	**CE**	**CD**	**BE**	**AE**	**E**	**Furans**	**LMWPC**	**Total phenolics**	
	**Fe**^**+2 **^**(mg/L)**	**H**_**2**_**O**_**2 **_**(mg/L)**		**UV**	*			**O**_**3 **_**(mg/L)**	*		*		*	*	**pH**				
A1	**2 (50)**	**2 (1000)**	2	**2 (present)**	2	2	1	**2 (500)**	2	2	1	2	1	1	**1 (pH 3)**	44	70	35	7
A2	**2 (50)**	**2 (1000)**	2	**2 (present)**	2	2	1	**1 (0)**	1	1	2	1	2	2	**2 (pH 8)**	3	23	1	49
**A3**	**2 (50)**	**2 (1000)**	2	**1**	1	1	2	**2 (500)**	2	2	1	1	2	2	**2 (pH 8)**	52	95	40	99
A4	**2 (50)**	**2 (1000)**	2	**1**	1	1	2	**1 (0)**	1	1	2	2	1	1	**1 (pH 3)**	11	16	−10	27
**A5**	**2 (50)**	**1 (0)**	1	**2 (present)**	2	1	2	**2 (500)**	2	1	2	2	1	2	**2 (pH 8)**	55	96	47	98
A6	**2 (50)**	**1 (0)**	1	**2 (present)**	2	1	2	**1 (0)**	1	2	1	1	2	1	**1 (pH 3)**	14	16	4	29
A7	**2 (50)**	**1 (0)**	1	**1**	1	2	1	**2 (500)**	2	1	2	1	2	1	**1 (pH 3)**	33	60	43	50
A8	**2 (50)**	**1 (0)**	1	**1**	1	2	1	**1 (0)**	1	2	1	2	1	2	**2 (pH 8)**	−3	11	−7	56
A9	**1 (0)**	**2 (1000)**	1	**2 (present)**	1	2	2	**2 (500)**	1	2	2	2	2	1	**2 (pH 8)**	50	95	41	66
A10	**1 (0)**	**2 (1000)**	1	**2 (present)**	1	2	2	**1 (0)**	2	1	1	1	1	2	**1 (pH 3)**	18	19	−12	6
A11	**1 (0)**	**2 (1000)**	1	**1**	2	1	1	**2 (500)**	1	2	2	1	1	2	**1 (pH 3)**	41	64	44	4
A12	**1 (0)**	**2 (1000)**	1	**1**	2	1	1	**1 (0)**	2	1	1	2	2	1	**2 (pH 8)**	−4	23	4	88
A13	**1 (0)**	**1 (0)**	2	**2 (present)**	1	1	1	**2 (500)**	1	1	1	2	2	2	**1 (pH 3)**	40	63	42	20
A14	**1 (0)**	**1 (0)**	2	**2 (present)**	1	1	1	**1 (0)**	2	2	2	1	1	1	**2 (pH 8)**	5	8	−11	82
A15	**1 (0)**	**1 (0)**	2	**1**	2	2	2	**2 (500)**	1	1	1	1	1	1	**2 (pH 8)**	54	95	44	79
A16	**1 (0)**	**1 (0)**	2	**1**	2	2	2	**1 (0)**	2	2	2	2	2	2	**1 (pH 3)**	−2	5	−6	32

* Columns used in error estimation. ** Percentage in relation to that obtained in the semi-synthetic medium fermentation (Q_P_ = 0.36 g/L.h).

Taguchi L_16_ experimental array, with different conditions of treatment by homogeneous AOPs evaluated, and responses: reduction of furans, low molecular weight phenolic compounds (LMWPC) and total phenols as well as the volumetric ethanol productivity by *Pichia stipitis* (expressed as a percentage relative to productivity obtained through the same conditions in semi-synthetic medium fermentation, Q_P_ = 0.36 g/L.h). The standard deviation was less than 5%.

In essentially every trial of the experimental design, changes in the concentration of total phenolic compounds, furans and LMWPC were observed. These findings indicate that this response is strongly influenced by these variables. The greatest reduction in the total phenolic concentration (35-47%) occurred in those treatments performed in the presence of ozone (assays A1, A3, A5, A7, A9, A11, A13 and A15). These results suggest that ozone imparts a great influence on the reduction of total phenolics in hemicellulosic hydrolysates. Similar to the observed reduction in phenolics, the highest reductions in total furan (33 to 55%) and LMWPC (60 to 96%) concentrations were observed in assays containing ozone (A1, A3, A5, A7, A9, A11, A13 and A15). Among the ozone-containing assays, those with basic pH (A3, A5, A9 and A15) showed reductions of approximately 53% and 95% for furans and LMWPC, respectively. This behavior demonstrates a positive interaction between the alkaline pH and the presence of ozone, resulting in favorable conditions for the removal of phenols and furan. Phenolic compounds become more susceptible to oxidative degradation at alkaline pH. In basic conditions phenolate ions are formed by the dissociation of the hydroxyl proton from the benzene ring, thereby increasing the electron density of the aromatic ring, which becomes more susceptible to oxidation [36].

In the present study, we demonstrate that AOPs preferentially remove aromatic compounds, such as furans and phenolic compounds, and do not interfere with the sugar concentration. In fact, some authors suggest that molecular structures with high electron densities, such as aromatic rings, can be preferentially oxidized by such processes [29,37]. The processes of Fenton (characterized by the presence of Fe^+2^ and H_2_O_2_ at acidic pH - assay A04), UV-peroxide (assay A10), and photolysis (assay A14) have been shown to reduce the levels of furans (5-18%) and LMWPC (8-19%) present in the hydrolysate. However, these observed reductions were mild compared with those shown in other assays of the experimental design.

### Evaluation of the treated hydrolysate fermentability by *Pichia stipitis*

**Figure 1:**
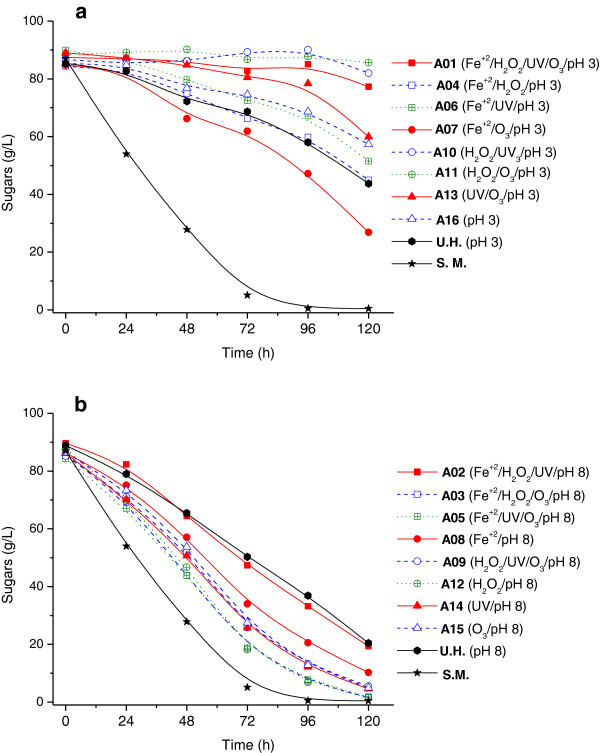
**Sugar consumption by *Pichia stipitis *in the hydrolysate treated in different conditions of experimental design.** Sugar consumption during the fermentations by *P. stipitis* in semi-synthetic medium (S.M.), untreated rice straw hemicellulosic hydrolysate (U.H.) and the hydrolysate treated by AOPs homogeneous under different conditions of the experimental design, where: (**a**) treatments performed at pH 3, (**b**) treatments performed at pH 8. The standard deviation was less than 5% of the mean values of sugars consumption

**Figure 2:**
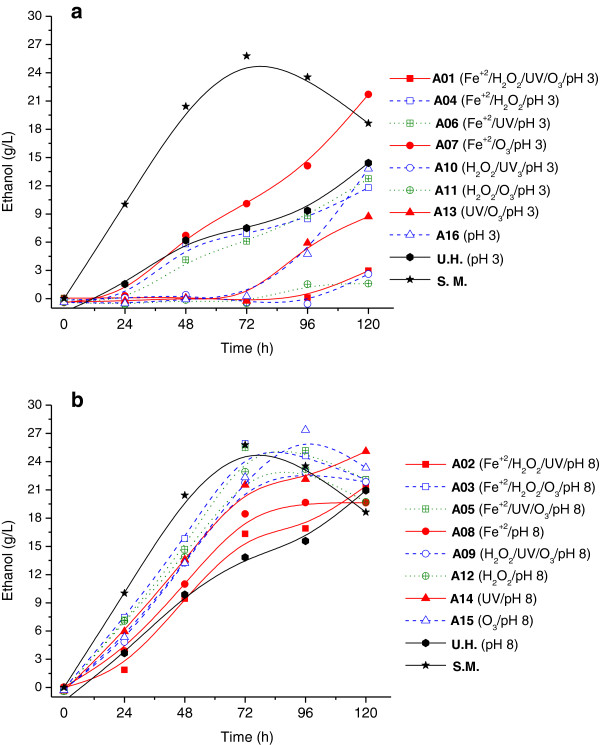
**Ethanol production by *Pichia stipitis *in hydrolysate treated in different conditions of experimental design.** Consumption of sugars (xylose and glucose in the proportion of 6:1) during the fermentations by *P. stipitis* in semi-synthetic medium (S.M.), untreated rice straw hemicellulosic hydrolysate (U.H.) and the hydrolysate treated by AOPs homogeneous under different conditions of the experimental design, where: (**a**) treatments performed at pH 3, (**b**) treatments performed at pH 8. The standard deviation was less than 5% of the mean values of ethanol production

The profiles of sugar consumption and ethanol production by *Pichia stipitis* in rice straw hemicellulosic hydrolysates (containing approximately 90 g/L sugar in a 6:1 proportion of xylose and glucose) after AOP detoxification are shown in Figures 1 and 2. There were a large number of assays performed; thus, to better understand the data, the experiments were divided into two groups. The hydrolysates treated in acidic medium (initial pH 3) were classified as group (a), and those treated in basic medium (initial pH 8) were designated as group (b). Fermentation in the hydrolysates of groups (a) and (b) was compared. In addition, the fermentation profiles with semi-synthetic medium and an untreated hydrolysate were included as controls.

Figure 1 shows the consumption of sugars (glucose and xylose) by the yeast *P. stipitis* in hydrolysates treated with different conditions according to our experimental design. In general, the pH was an important variable affecting hydrolysate fermentability. The use of alkaline conditions for hydrolysate treatment favored both sugar consumption and ethanol production. Yeast cultured in hydrolysates treated with acidic pH (Figure 1a), with the exception of treatment A7, did not exhibit improved sugar consumption compared to untreated hydrolysates. For example, yeast cultivated in assays A4, A6, A13 and A16 exhibited similar or lower sugar consumption compared to those cultivated in the untreated hydrolysate whereas in assays A1, A10 and A11 any sugar consumption was observed. On contrary, in the hydrolysates treated with alkaline conditions (Figure 1b) the sugar utilization was favored. In assays A3, A5 and A12, 90% of the sugars were utilized by the yeast in 96 hours, and in assays A9, A14 and A15, approximately 82% of the sugars were consumed during the same time. Although this group of assays showed a lower sugar intake than fermentation in semi-synthetic medium (100% in 96 hours), there was a considerable increase in consumption compared to fermentation in the untreated hydrolysate (51% in 96 hours).

Regarding ethanol production (Figure 2), it appears that among the treatments carried out in acid pH (Figure 2a), only assay A7 favored ethanol production by *P. stipitis*. Under this condition, the yeast produced approximately 22 g/L of ethanol after 120 hours, whereas the untreated hydrolysate produced only 15 g/L during the same fermentation time. This increase in ethanol production corresponds to a 50% increase due to treatment. Similar to fermentation in the untreated hydrolysate, the yeast from assays A4 and A6 yielded only 13 g/L of ethanol after 120 hours. On the other hand, in assays A13 and A16, the yeast underwent a long lag phase of growth, and as a consequence, the ethanol production started after 72 hours of fermentation and reached concentrations of 9 g/L and 14 g/L, respectively. The lowest concentrations of ethanol, nearly 3 g/L (after 120 hours), were observed in assays A1, A10 and A11 in which the yeast were not able to efficiently consume sugars (Figure 1a).

Compared to untreated hydrolysates, yeast showed an increase in ethanol production in all treatments performed in alkaline conditions (Figure 2b). The hydrolysates treated in assays A3 and A5 produced ethanol up to 26 g/L after 72 hours of fermentation. This concentration is almost the same as that observed in semi-synthetic medium and is approximately twice the value found for the untreated hydrolysate cultivated for the same fermentation time (approximately 14 g/L).

In assays A9, A12, A14 and A15, ethanol concentrations near 23 g/L were reached after 72 hours of cultivation. This concentration corresponds to a 60% increase in ethanol production over the untreated hydrolysate fermented for the same length of time. Among the treatments carried out in alkaline medium, assays A2 and A8 had the lowest ethanol concentration, showing an ethanol production profile very similar to that of the untreated hydrolysate fermented for 48 hours. However, after 72 hours of fermentation, A2 and A8 assays showed an increase in ethanol concentration of 18% and 33%, respectively.

In general, the hydrolysates treated with AOPs under acidic conditions (pH 3) exhibited reduced sugar consumption and ethanol production with respect to the untreated hydrolysate, even the assays A1, A11 and A13, which showed considerable reduction in the concentration of total phenols, LMWPC and furans. This result indicates that the degradation of these compounds may have resulted in more toxic products that may have been more harmful to the fermentation process. On contrary, a reduction in hydrolysate toxicity occurred in the treatments performed at a basic pH (pH 8). This trend may be related to specific pH-dependent mechanisms of attack of the hydroxyl radicals, which may lead to various degradation products. An example of these different degradation mechanisms is the formation of phenolate ions by the dissociation of a proton from the aromatic ring's hydroxyl, which occurs under alkaline conditions. The formation of these ions leads to an increase in the electron density of the aromatic ring, making it more susceptible to oxidation [36]. As with the hydroxyl group of the aromatic ring, different groups attached to the ring can dissociate at different pH values, thereby affecting the highly electron-rich regions of the molecules [38]. These variations in electronegativity for aromatic compounds can interfere with the orientation of hydroxyl radical attack, which is electrophilic in nature [29].

**Figure 3:**
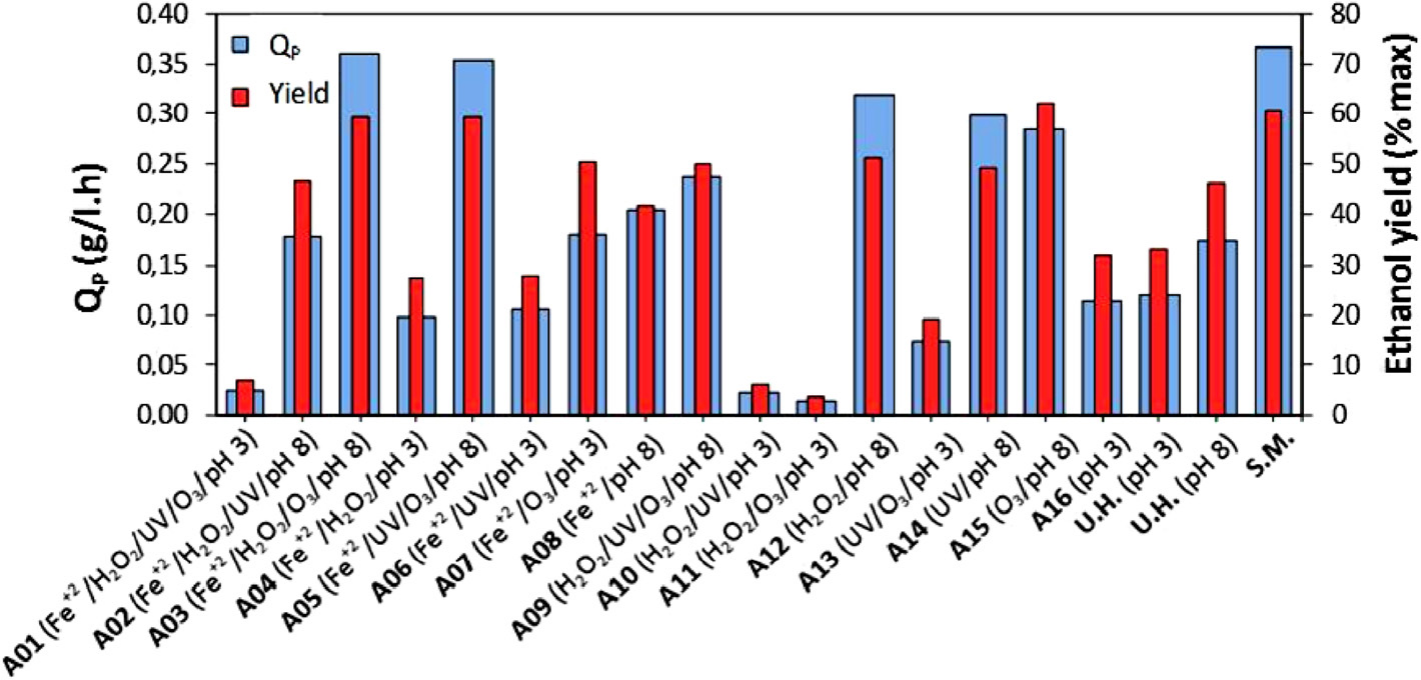
**Fermentation parameter of *Pichia stipitis *cultivation in the different conditions of the experimental design.** Ethanol volumetric productivity (□) and ethanol yield expressed as % of theoretical maximum based on total content of fermentable sugars (□), observed in fermentations by *P. stipitis* in semi-synthetic medium, untreated rice straw hemicellulosic hydrolysate and the hydrolysate treated by AOPs homogeneous under different conditions of the experimental design

Figure 3 shows the ethanol volumetric productivity (Q_P_) and ethanol yield (expressed as% of theoretical maximum based on total content of fermentable sugars) for fermentation by *P. stipitis* in an untreated hydrolysate, in hydrolysates treated by AOPs and in a semi-synthetic medium. The values of Q_P_ varied from 0.02 to 0.36 g/L.h, demonstrating that the different treatment conditions greatly influenced this parameter. The highest values for volumetric productivity (approximately 0.36 g/L.h) were achieved in assays A3 and A5, which yielded up to 98% of the productivity obtained using a semi-synthetic medium. These two assays also showed elevated levels for the removal of total phenols (up to 47%), LMWPC (up to 96%) and furans (up to 55%). Tests A1, A10, A11 and A13 had values of Q_P_ less than 0.07 g/L.h, which is less than that observed from the untreated hydrolysate. This low Q_P_ may be due to the formation of more toxic compounds. Similar to the behavior observed for Q_P_, the values of ethanol yield showed a large variation (4 a 60% of maximum theoretical based on substrate content). In general, the fermentations of hydrolysate treated in pH 3 showed the same or lower ethanol yields when compared to untreated hydrolysate, whereas the treatments carried in alkaline conditions (pH 8) resulted in improvements on ethanol yields. The highest values for ethanol yield (approximately 60% of theoretical maximum ethanol) were achieved in assays A3, A5 and A15, which were similar to that obtained using a semi-synthetic medium.

These results demonstrate that the AOPs mediated detoxification of rice straw hemicellulosic hydrolysates can affect the hydrolysate fermentability to different degrees. The greatest impact of treatment occurred on the volumetric productivity of the fermentation process.

### Statistical analysis

We used a statistical analysis to identify the most important factors to influence the detoxification of rice straw hemicellulosic hydrolysates by AOPs. In this analysis, we evaluated the effects of Fe^+2^ (A), H_2_O_2_, (B), UV irradiation (C), O_3_ (D) and pH (E). The following combinations of factors were also evaluated: AB (Fe^+2^/H_2_O_2_ – the Fenton process), BC (H_2_O_2_/UV), DE (O_3_/pH), BD (H_2_O_2_/O_3_) and CD (UV/O_3_). The observed responses were the percent reduction in the concentrations of furans (furfural and HMF), LMWPC and total phenolics and changes in the ethanol volumetric productivity were measured for the fermentation of *P. stipitis* in hydrolysates from each treatment.

The statistical significance of the main effects and their interactions on the different responses were verified using an analysis of variance (ANOVA) test, presented in Table 3. According to the ANOVA (design L_16_), the percentages of variation in the concentrations of furans, total phenolics and LMWPC showed high correlation coefficients (97, 99 and 95%, respectively). Thus, the observed variation can be explained by the factors that were evaluated. Ozone treatment was responsible for more than 87% of all the observed variation showing a positive effect on the removal of furans, total phenolics and LMWPC.

**Table 3:** Analysis of variance for the response evaluated in the experimental design

**Source of variation**	**Taguchi L**_**16**_				**Taguchi L**_**8**_
	**Furans reduction**	**LMWPC**	**Total phenolics reduction**	**Q**_**P **_(%)****	**Q**_**P **_(%)****
A - Fe^+2^	0.1	0.1	0.0	0.6	0.8
B - H_2_O_2_	0.3	0.8**	0.1	3.8	0.9
AB	0.0	0.0	0.6	0.0	0.0
C - UV	1.9**	0.2	0.0	2.3	3.9
BC	0.2	0.0	0.3	4.0	71.8**
DE	7.3*	4.4*	0.0	2.4	-
D - O_3_	87.8*	87.9*	95.5*	1.0	22.2***
BD	0.1	0.3	0.3	0.6	-
CD	0.6	0.0	0.0	0.0	-
E - pH	0.1	5.9*	0.2	74.6*	-
R^2^ (%)	97.0	99.0	95.5	74.6	94.0

* Significant at 99% confidence level; ** Significant at 95% confidence level; *** Significant at 90% confidence level. **** Percentage in relation to that obtained in the semi-synthetic medium fermentation (Q_P_ = 0.36 g/L.h).

Analysis of variance of the main effects and of its interactions factors on the responses: reductions concentrations of furans, low molecular weight phenolic compounds (LMWPC) and total phenolics, besides of the ethanol volumetric productivity by *Pichia stipitis* (Q_P_), for the original design (Taguchi L_16_) and ethanol volumetric productivity to planning referent to process at alkaline pH only (Taguchi L_8_).

Statistical analysis showed that for furan reduction, O_3_ (D) and the combination of O_3_ and pH (DE) were significant with confidence level of 99%, and UV radiation (C) was significant with confidence level of 95%. For LMWPC reduction, O_3_ (D), pH (E) and the combination of O_3_ and pH (DE) were considered significant with a confidence level of 99%. In addition, the H_2_O_2_ influence (factor B) was also significant, at a level of 95% confidence. Regarding the total phenolics, only ozone was significant with a confidence level of 99%. All these significant variables showed positive effects on furans, LMWPC and total phenolics removal.

The volumetric productivity (Q_P_) of a fermentation process is an important parameter because it relates the concentration of the product formed with the time required for production. This results in an interesting basis for comparisons. To evaluate the influence of different hydrolysate treatments on yeast fermentability, the relationship between the ethanol volumetric productivity was used as the response variable. This response was compared to the productivity obtained from fermentation in semi-synthetic medium under the same conditions (100% corresponds to the same productivity obtained using semi-synthetic medium, Q_P_ = 0.36 g/L.h) (Table 2).

The ANOVA (design L_16_), for the Q_P_ (Table 3) showed a reasonable correlation coefficient (75%). Seventy four percent of the observed variation could be attributed to Factor E. The importance shown by this factor is explained by the fact that almost all treatments under acidic conditions led to low hydrolysate fermentability. The opposite effect was observed for the treatments carried out under alkaline conditions. Thus, increasing the pH value had a positive effect on ethanol volumetric productivity. To further analyze the influence of the other treatment conditions on the hydrolysate fermentability, the experimental design L_16_ assays were rearranged into a new experimental matrix, L_8_. In the new matrix, only the tests performed under alkaline conditions were considered.

There was a high correlation coefficient (94%) in the variance analysis (ANOVA) of matrix L_8_. The high correlation demonstrates the significance of the particular factors evaluated for their influence on ethanol volumetric productivity. Variable D (O_3_) and the combination BC (H_2_O_2_/UV) were significant at a confidence level of 90 and 95%, respectively. Only variable D (O_3_) had a positive effect on increasing the hydrolysate fermentability. The combination BC (H_2_O_2_/UV) showed a negative effect, indicating that the use of UV radiation in the absence of H_2_O_2_ (or H_2_O_2_ in the absence of UV) can collaborate with the process because these conditions represent an adjustment of the interaction term BC at its lowest level (level 1).

The statistical analysis indicate that, in the experimental evaluated range, ozonation in alkaline medium (pH 8) in the presence of H_2_O_2_ (treatment A3) or in the presence of UV radiation (treatment A5) were the most effective methods for the hydrolysate detoxification that in turn increase the yeast fermentability on rice straw hemicellulose hydrolysates. Further analysis such as scanning UV spectroscopy, infrared spectroscopy and were performed using hydrolysate treated according the assay A3.

### Ultraviolet spectra profiles of the hydrolysates treated by AOPs

Absorbance within the ultraviolet spectrum is a technique that can provide information regarding the presence of phenolic compounds derived from lignin and furans resulting from the degradation of carbohydrates. This technique permits one to evaluate parameters including changes in concentration or even changes in the chemical structure of UV-absorbable molecules [2,39-41].

The differential spectrum technique is a methodology that helps interpret ultraviolet spectrum data. This technique can provide important information about the presence of ionizable groups, such as the phenolic hydroxyl group. These spectra are obtained by determining the scan profile of a sample in basic solution and zeroing the same sample in an acidic or neutral solution [2,39].

The differential spectrum for phenolic hydroxyls exhibits three characteristic peaks with maxima near 250, 300 and 350–400 nm. These peaks differ mainly due to the presence of phenolic hydroxyl groups that are conjugated (or not) with the aromatic carbonyl. The peak with a maximum between 350–400 nm is attributed to compounds having aromatic ring-mediated conjugation between the hydroxyl and the carbonyl, the peak with a maximum at approximately 300 nm is characteristic of non-conjugated hydroxyl groups and the peak with a maximum near 250 nm is attributed to the presence of both conjugated and non-conjugated structures [41,42].

**Figure 4:**
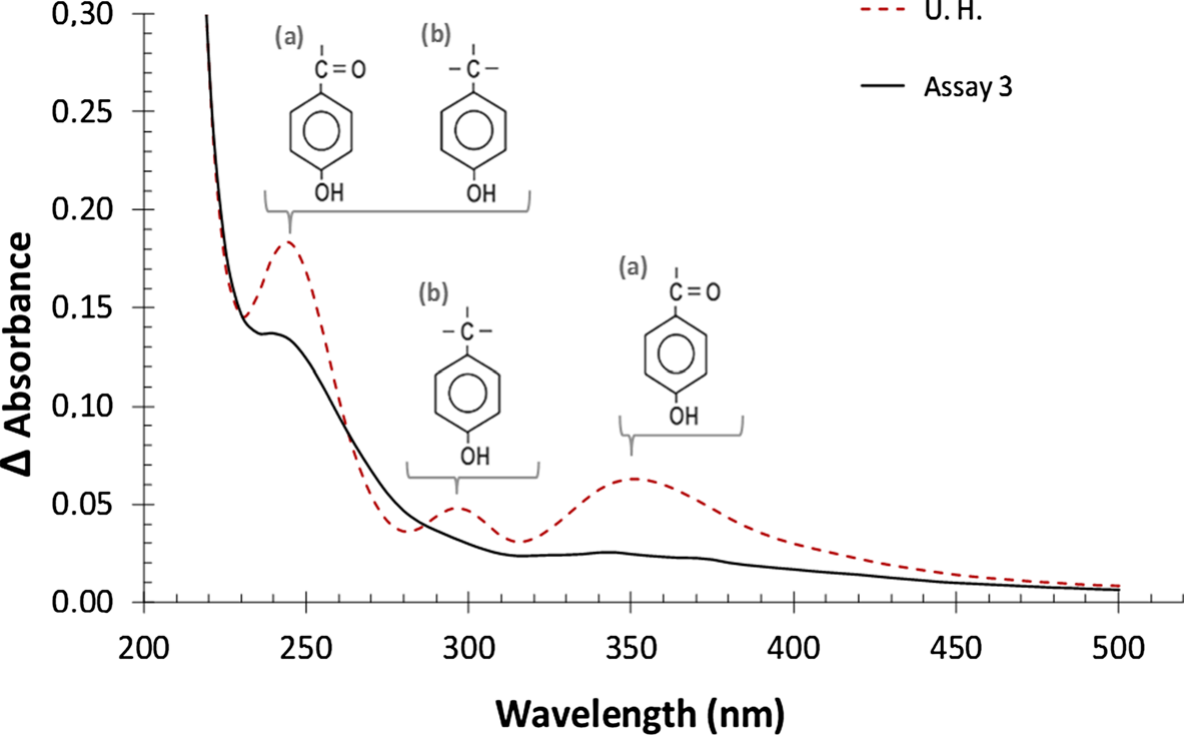
**Ultraviolet differential spectrum profile before and after the detoxification treatment.** Ultraviolet differential spectrum of the rice straw hemicellulosic hydrolysate before (- - -) and after (—) treatment by AOPs homogeneous in conditions of assay A3 of the experimental design. Phenolic hydroxyl groups conjugated (**a**) and non-conjugated (**b**) with the carbonyl through the aromatic ring

Figure 4 shows the differential spectrum of the untreated hydrolysate and the hydrolysate treated by AOPs in the A3 assay. In the differential spectrum of the untreated RSHH (Figure 4), there were three maxima at 245, 295 and 350 nm, indicating the presence of conjugated and non-conjugated phenolic hydroxyl groups. Variations were observed between the differential spectra of the treated hydrolysate from assay A3 and the untreated hydrolysate. After treatment, there were reductions in the three maxima (245, 295 and 350 nm). These results demonstrate that the number of hydroxyl groups present in phenolic compounds was reduced upon treatment. Such reductions are related to changes in the aromatic hydroxyl groups or to ring cleavage resulting from the oxidative processes used.

### Evaluation of the infrared spectrum of the hydrolysates treated by AOPs

**Figure 5:**
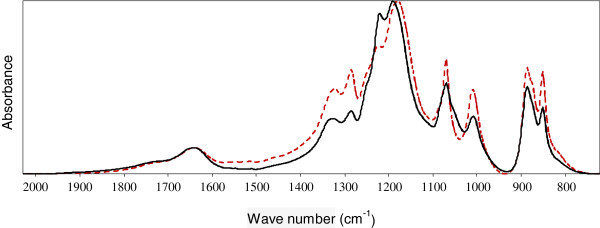
**Infrared spectrum profile before and after the detoxification treatment.** Infrared spectrum of the rice straw hemicellulosic hydrolysate before (- - -) and after (—) treatment by AOPs homogeneous in conditions of assay A3 of the experimental design

The infrared spectra (700–2000 cm^-1^) of the rice straw hydrolysate media before and after treatment with the A3 assay conditions are shown in Figure 5. Each spectrum was standardized against the most intense signal (at approximately 1180 cm^-1^). The infrared spectrum of the untreated hydrolysate exhibited signals at 1720, 1640, 1326, 1286, 1223, 1178, 1070, 1008, 886, and 851 cm^-1^.

The treatment conditions employed in the A3 assay were able to change the signal intensities at multiple absorption peaks in the infrared spectrum of the treated hydrolysate. After treatment, the signal intensities at 1326, 1286, 1070, 1008, 886 and 851 cm^-1^ were reduced, there was an increase in the relative intensity of the absorption peak at 1223 cm^-1^, and shifts were observed at the 1178 cm^-1^ signal for the 1193 cm^-1^ wave number.

In general, the changes observed in the spectra of the treated hydrolysates demonstrate that the AOPs acted on the compounds derived from lignin, providing variations in the signal intensities at different wavelengths. Due to the complex mixture of different compounds, the signal changes may be associated with the selective degradation or removal of certain components or with chemical modifications and changes to the functional groups as a result of the treatments.

## Conclusions

The AOPs treatments were able to preferentially removed compounds with high electron densities, such as furans and phenolic compounds, without affecting the sugar concentration in the rice straw hemicellulose hydrolysates. Among the different conditions of treatment with AOPs evaluated, ozonation in alkaline medium (pH 8) in the presence of H_2_O_2_ (treatment A3) or UV radiation (treatment A5) were the most effective for hydrolysate detoxification and had a positive effect on increasing the yeast fermentability of rice straw hemicellulose hydrolysate. Under these conditions, the higher removal of total phenols (above 40%), LMWPC (above 95%) and furans (above 52%) were observed. In addition, the ethanol volumetric productivity by *P. stipitis* was increased in approximately twice in relation the untreated hydrolysate. Based on the data, the AOPs can be considered promising methods to detoxify hydrolysates of lignocellulosic biomass for applications in bioconversion processes, and therefore is a field that deserves more attention.

## Materials and methods

### Preparation of rice straw hemicellulosic hydrolysate

Rice straw was collected from fields near Lorena city, São Paulo state, Brazil. The material was naturally dried in the sun until approximately 10% moisture content, milled to attain particles of about 1 cm in length and 1 mm in thickness, and stored. Hydrolysate was prepared by dilute acid hydrolysis as described by Roberto, Mussatto and Rodrigues [43]. Hydrolysis was performed in 50 L reactor, heated by electric resistance and agitated by rotation on own axis. The reaction was carried at 120°C for 30 minutes, 3 rpm and the rice straw was impregnated with sulfuric acid solution (100 mg H_2_SO_4_/g dry matter) in a 1:10 (g/mL) dry matter:acid solution ratio. After hydrolysis, the residual solid material was separated by centrifugation and the liquid phase (hemicellulosic hydrolysate) was submitted to a vacuum concentration process at 65°C, in a 30 L stainless steel evaporator, aiming to increase the xylose concentration about 120 g/L. The concentrated hydrolysate was stored at 4°C for later use.

### Hydrolysate treatment

The rice straw hemicellulosic hydrolysate was submitted to different treatments conditions by homogeneous AOPs (Fenton, Photo-Fenton, UV/H_2_O_2_, O_3_ and UV/O_3_). The main characteristic of this group of AOPs is that all the chemical species responsible for radical hydroxyl generation are soluble in liquid phase. Experiments were carried out using an array of Taguchi L_16_ (Table 2), which consisted of 16 assays in which the variables: presence of Fe^2+^, H_2_O_2_, UV irradiation, O_3_, and pH were studied at two levels (1 and 2), as shown in Table 4. The observed responses were the change in sugar concentration, reducing the toxic compounds concentration and the fermentability of the treated hydrolysate.

**Table 4:** Values and levels of variables used in the experimental design

**Variables**	**Level**	
	**1**	**2**
Fe ^2+^ (A)	Not added*	Addition of 50 mg/L
H_2_O_2_ (B)	0 mg/L	1000 mg/L
UV-C (C)	Absence	Presence
O_3_ (D)	0 mg/L	500 mg/L**
pH (E)	3.0	8.0

* Only the amount of Fe^2+^ present in the hydrolysate. ** Ozone dissolution of 2.5 mg/minute (produced from a flow of 1 l/minute of O_2_) for 30 minutes to a volume of 150 mL hydrolysate.

Values and levels of variables used in the Taguchi L_16_ experimental design to evaluate the treatment of hydrolysate by homogeneous advanced oxidative process.

The treatments were conducted in a glass reactor with a capacity of 150 mL, equipped with water refrigeration and magnetic stirrer. Irradiation was performed using a germicidal lamp (UV-C) of 4 W of power, which is submerged in the reaction medium through a quartz bulb. Ozone was produced by conversion of O_2_ to O_3_ by corona effect, being a device used Ozone Generator Ozonebras, Z30 model with an output of 60 W, at a flow rate of 1 L/min of oxygen. All treatments were carried out for 30 minutes at a controlled temperature of 30°C, under conditions which varied according to experimental design described below. Before the realization of the experimental design the concentrated rice straw hemicellulosic hydrolysate (Table 1) had the pH adjusted to values corresponding to the levels of the experimental design (pH 8 and 3) with NaOH 10 mol/L. The precipitate formed after each pH change was removed by centrifugation (1000 xg for 15 minutes), and then xylose concentration in the hydrolysate was adjusted to approximately 90 g/L.

### Fermentative process

Fermentations were carried out in semi-synthetic or hydrolysate medium. Before the use of the rice straw hemicellulosic hydrolysate as a fermentation medium, its pH was changed to pH 5.5 with NaOH 10 mol/L or H_2_SO_4_ 10 mol/L as required. After each change of pH, the hydrolysate was centrifuged at 1000 xg for 15 minutes to remove solid residue.

### Inoculum preparation

*Pichia stip*itis NRRL Y-7124 was the microorganism used in the experiments. Cultures of this yeast were maintained on malt extract agar slants at 4°C. For fermentations in semi-synthetic medium, inoculum was prepared by transfer of cells of the yeast in the maintenance medium to 125 mL Erlenmeyer flasks containing 25 mL of the medium composed by (g/L): xylose (20.0), glucose (3.3), arabinose (3.3), urea (2.3), MgSO_4_.7H_2_O (1.0) and yeast extract (3.0). For fermentations in hydrolysate, the inoculum was prepared also in the hydrolysate, whose concentrations of sugars were adjusted by dilution to (g/L): xylose (20.0), glucose (3.3), and arabinose (3.3); without the addition of yeast extract (3.0). The inoculated flasks were incubated in a rotary shaker at 30°C under stirring at 200 rpm for 24 hours. After this time the cells were recovered by centrifugation (1100 xg for 20 minutes) and resuspended in sterile distilled water in order to obtain a concentrated suspension of cell which was used as inoculum.

### Media and fermentation conditions

The semi-synthetic medium was composed by (g/L): xylose (70.0), glucose (12.0), arabinose (12.0), urea (2.3), MgSO_4_.7H_2_O (1.0) and yeast extract (3.0). The hydrolysate based medium was composed by (g/L): xylose (70.0), glucose (12.0), arabinose (12.0) with addition of yeast extract (3 g/L). The fermentations were carried out in 125 mL Erlenmeyer flasks containing 50 mL of fermentation medium and inoculated with 1 g/L of cells. The flasks were incubated in a rotary shaker at a temperature of 30°C and shaken at 200 rpm for 120 hours.

### Analytical methods

Glucose, xylose, arabinose, acetic acid, and ethanol concentrations were determined by high performance liquid chromatography (HPLC) in Waters chromatograph equipped with a refractive index detector and a Bio-Rad Aminex HPX-87H column (300 × 7.8 mm). Operation conditions included: temperature of 45°C, 0.005 M sulfuric acid as eluent in a flow of 0.6 mL/min, and sample volume of 20 μL. The cellular growth was determined by measuring the fermentation broth at UV-spectrophotometric absorbance at 600 nm, which was correlated to a calibration curve (dry weight × optical density).

Furfural, hydroxymethylfurfural, vanillic acid, vanillin, *p-*coumaric acid, and ferulic acid were also determined by HPLC, using an UV detector (at 276 nm) and a ZORBAX Eclipse Plus C18 column (4.6 × 100 mm and 3.5 μm particle size) at room temperature, acetonitrile/water/acetic acid at a ratio of 88:11:1 as the eluent, a flow rate of 0.8 mL/min and sample volume of 20 μL. The concentration of total phenolic compounds was estimated by Folin-Ciocalteau method [44] using ferulic acid as standard. Ultraviolet spectrum of the hydrolysate was determined in Hitachi U-2000 spectrophotometer, in the range of 500–200 nm, with a pitch of 5 nm using quartz cuvettes. The determination of the spectrum was made with hydrolysate diluted with alkaline water (pH around 12), and distilled water used as a blank. The differential scanning spectra were obtained by determining the profile scan of the hydrolysate in alkaline media (pH 12), using as zero the same sample, but in acidic media (pH 2). The infrared spectra, 2000–700 cm^-1^ were determined in a spectrophotometer Perkin Elmer Spectrum One. The hydrolysate was submitted to a solid–liquid extraction in Sep-Pak C18 (Waters) for the separation of the phenolic compounds (retained on Sep-Pak), which was recovered and used to determine the infrared spectra. The samples were prepared by mixing one drop of the fraction recovered 100 mg of KBr, which was dried in desiccators under vacuum over P_2_O_5_. The dried samples were processed in KBr tablet compressing it at a pressure of 10 kgf.cm^2^. Inserts made of pure KBr were used as reference.

Ethanol yield was expressed as% of the ratio between ethanol concentration produced and the theoretical maximum ethanol based on total content of fermentable sugars. Ethanol volumetric productivity (Q_P_, g/L.h) was calculated as the ratio between the ethanol concentration (g/L) and the fermentation time (h).

## Abbreviations

AOPs: Advanced Oxidative Processes; RSHH: Rice Straw Hemicellulosic Hydrolysate; HMF: Hydroxymethylfurfural; HPLC: High performance liquid chromatography; LMWPC: Low molecular weight phenolic compounds; UV: Ultraviolet radiation; H2O2: Hydrogen peroxide; O3: Ozone; O2: Oxygen; H2SO4: Sulfuric acid; Fe2+: Iron divalent ion; NaOH: Sodium hydroxide; KBr: Potassium bromide; P2O5: Phosphorus pentoxide; ANOVA: Analysis of variance; QP: Ethanol volumetric productivity; SM: Semi-synthetic medium; UH: Untreated rice straw hemicellulosic hydrolysate.

## Competing interests

The authors declare that they no competing interests.

## Authors' contributions

JPAS participated in the design of the study, carried out the experiments, acquired the data and drafted the manuscript. LMC participated in the design of the study, co-conducted the experimental works and participated in the result interpretation. ICR participated in the design of the study, participated in the results interpretation and analysis, and critically revised the manuscript for important intellectual content. All authors read and approved the final manuscript.
